# Supplementary material for: Structural basis for the intracellular regulation of ferritin degradation
Source: Nat Commun. 2024 May 7;15:3802. doi: 10.1038/s41467-024-48151-1 (PMC11076521; doi:10.1038/s41467-024-48151-1)
Supplement: Supplementary file 1 — Supplementary Information [file 41467_2024_48151_MOESM1_ESM.pdf]

Supplementary Information

Structural basis for the intracellular regulation of ferritin degradation

Fabian Hoelzgen[1,2,$], Thuy T. P. Nguyen[3,$], Elina Klukin[2], Mohamed Boumaiza[4], Ayush K. Srivastava[4], Elizabeth Y. Kim[3], Ran Zalk[5], Anat Shar[5], Sagit Cohen-Schwartz [6], Esther G. Meyron-Holtz[7], Fadi Bou-Abdallah[4], Joseph D. Mancias[3,8,*], Gabriel A. Frank[2,5,6,*]

1. The Kreitman School of Advanced Graduate Studies, Marcus Family Campus, Ben-Gurion University of the Negev, Beer-Sheva, Israel
2. Department of Life Sciences, Marcus Family Campus, Ben-Gurion University of the Negev, Beer-Sheva, Israel
3. Division of Radiation and Genome Stability, Department of Radiation Oncology, Dana-Farber Cancer Institute, Harvard Medical School, Boston, Massachusetts, USA
4. Department of Chemistry, State University of New York at Potsdam (SUNY Potsdam), Potsdam, New York, USA
5. Ilse Katz Institute for Nanoscale Science & Technology, Marcus Family Campus, Ben-Gurion University of the Negev, Beer-Sheva, Israel
6. The National Institute for Biotechnology in the Negev – NIBN, Marcus Family Campus, Ben-Gurion University of the Negev, Beer-Sheva, Israel
7. Faculty of Biotechnology and Food Engineering, Technion, Haifa, Israel
8. Department of Radiation Oncology, Brigham and Women's Hospital, Harvard Medical School, Boston, Massachusetts, USA

$ These authors contributed equally to this work.

* Correspondence and requests for materials should be addressed to: Dr. Gabriel A. Frank and Dr. Joseph D. Mancias:
G.A.F. (email: frankg@bgu.ac.il)
J.D.M. (email: Joseph_Mancias@dfci.harvard.edu)

The PDF file includes:

Supplementary Figures
Supplementary Figures 1-7

Supplementary Tables
Supplementary Table 1

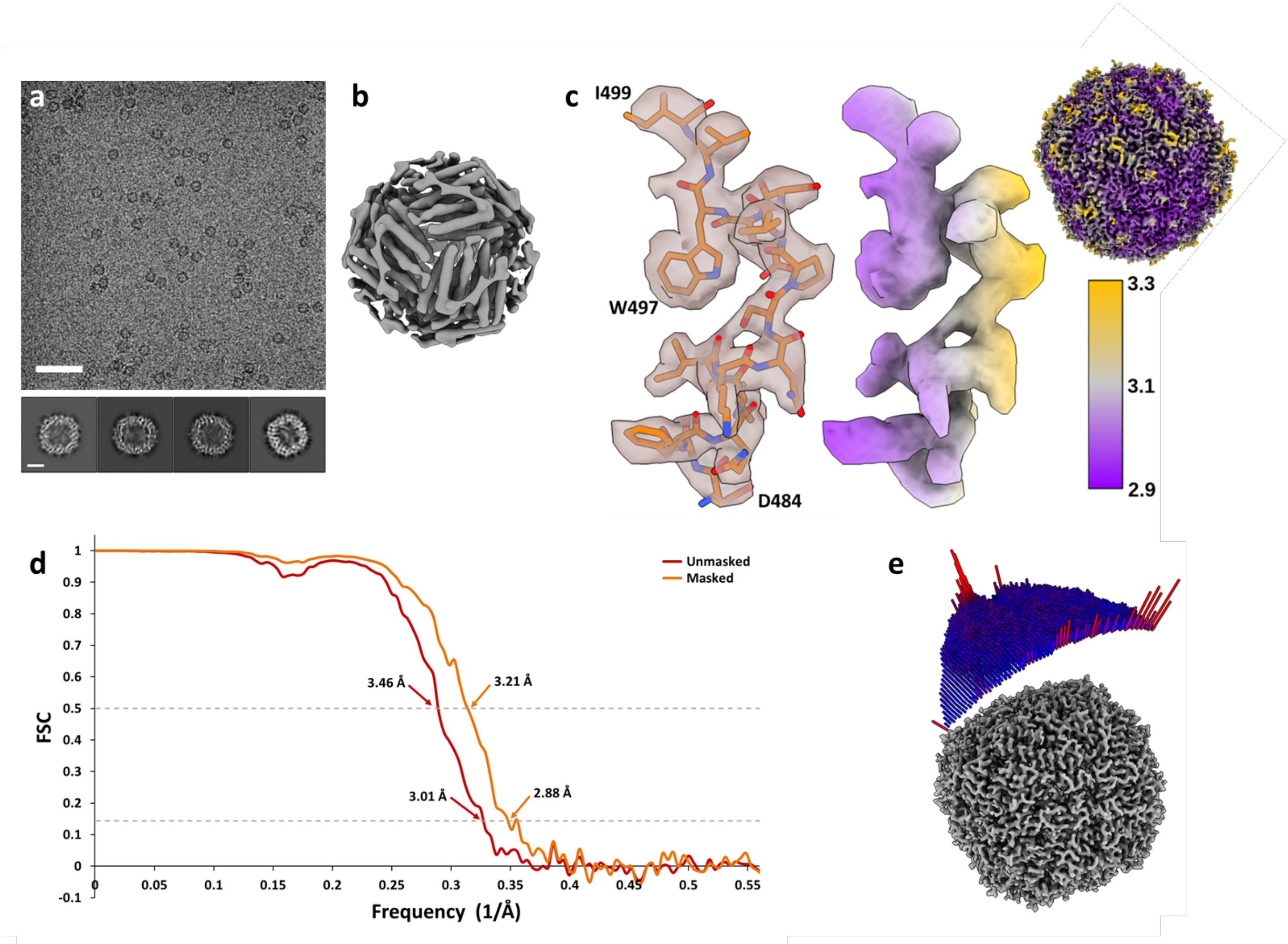

Supplementary Fig. 1 Summary of image processing of the NCOA4^{FB}·FTH1 complex datasets. (**a**) A representative segment from a micrograph of the "mix on grid" NCOA4^{FB}·FTH1 complex, showing rounded particles typical of ferritin nanocages (scale bar 50 nm), and 2D-class averages resulting from this dataset (scale bar 5 nm). (**b**) The selected 3D class average consisting of ~34 k particles used for further refinement. (**c**) A part of the electron density map surrounding the molecular model of NCOA4^{FB}, showing the overall fit and the local resolution assessment of this region, and the entire map (top right) represented as surface colors (color key is in Å). (**d**) FSC plot of the resulting 2.88 Å 3D-EM map of the NCOA4^{FB}·FTH1complex. (**e**) Histogram of the angular distribution of particles contributing to the final reconstructed map.

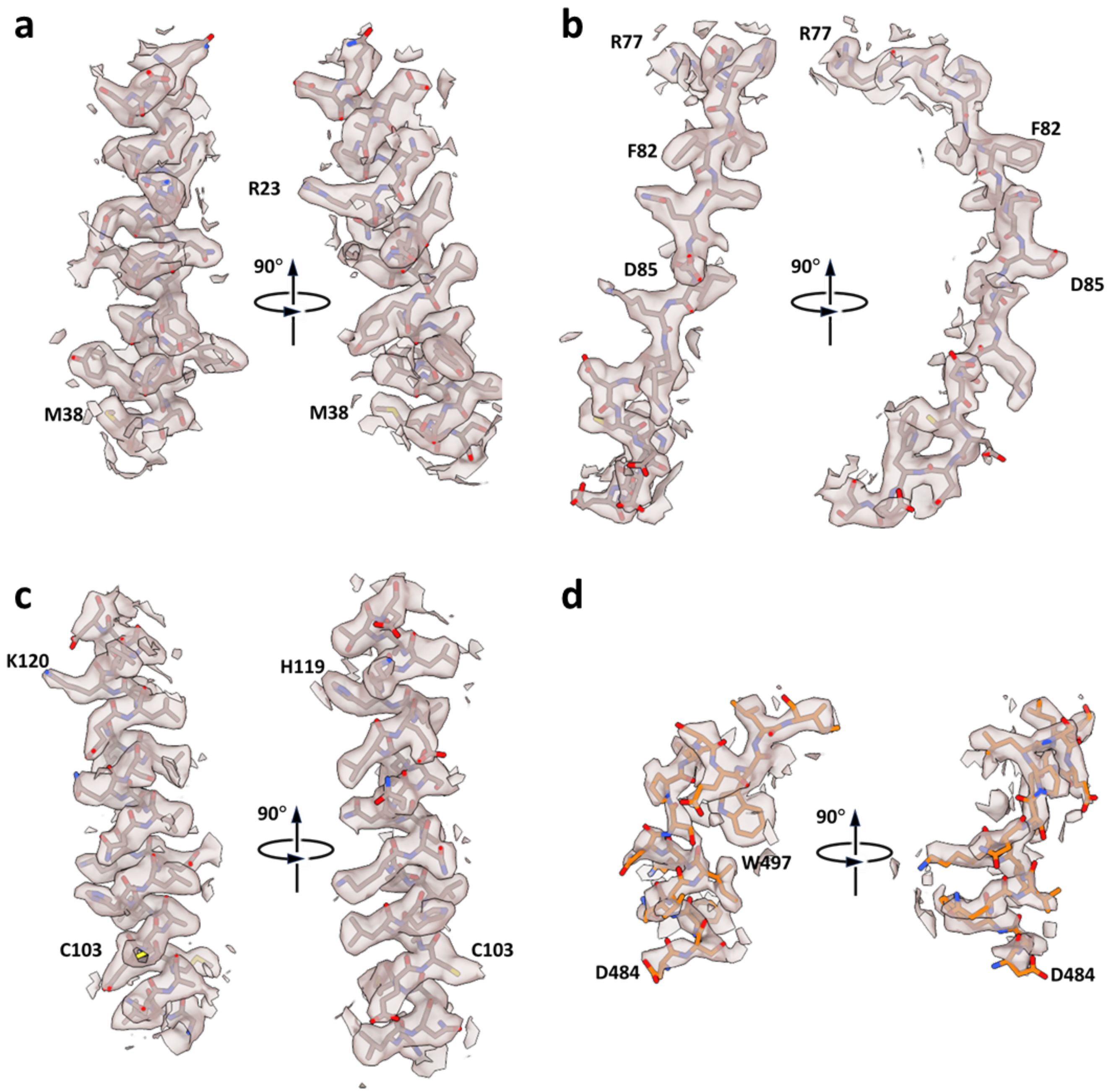

Supplementary Fig. 2 Prominent structural elements and their fit into the 3D-EM map. (**a**) FTH1 Helix a. (**b**) The b-c loop. (**c**) Helix c. (**d**) NCOA4^{FB}. The 3D-EM map in all panels is at the same contour level, amino acids from each segment are designated to guide the eye.

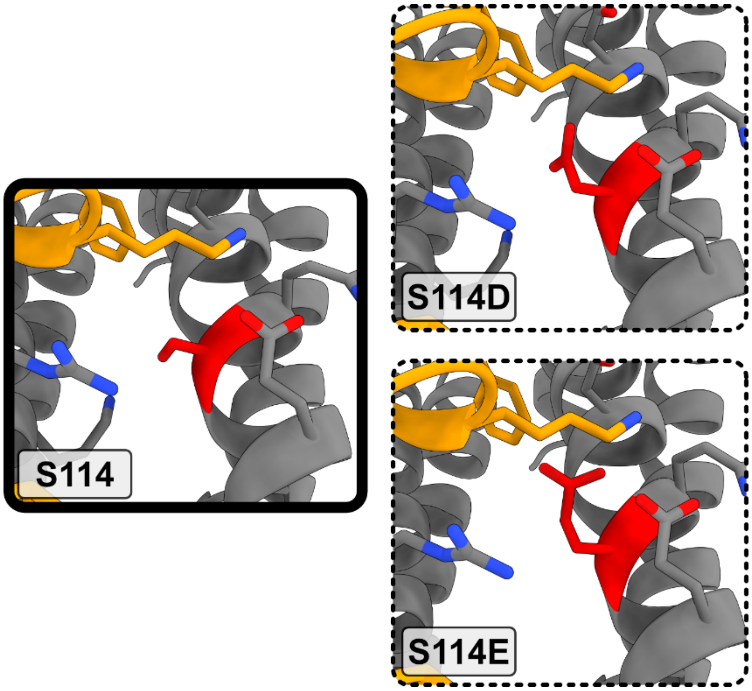

Supplementary Fig. 3 Visualization of the phosphomimetic mutations S114D and S114E. NCOA4 is orange, FTH1 is gray, and mutated amino acids are in red; dashed frames designate the presumed position of the amino acids after mutation. These phosphomimetic mutations were designed to test the effect of phosphorylation of S114 on the interaction of NCOA4 with FTH1. Structural analysis does not indicate a strong effect of either of these mutations. S114E may cause steric hindrance for NCOA4 binding.

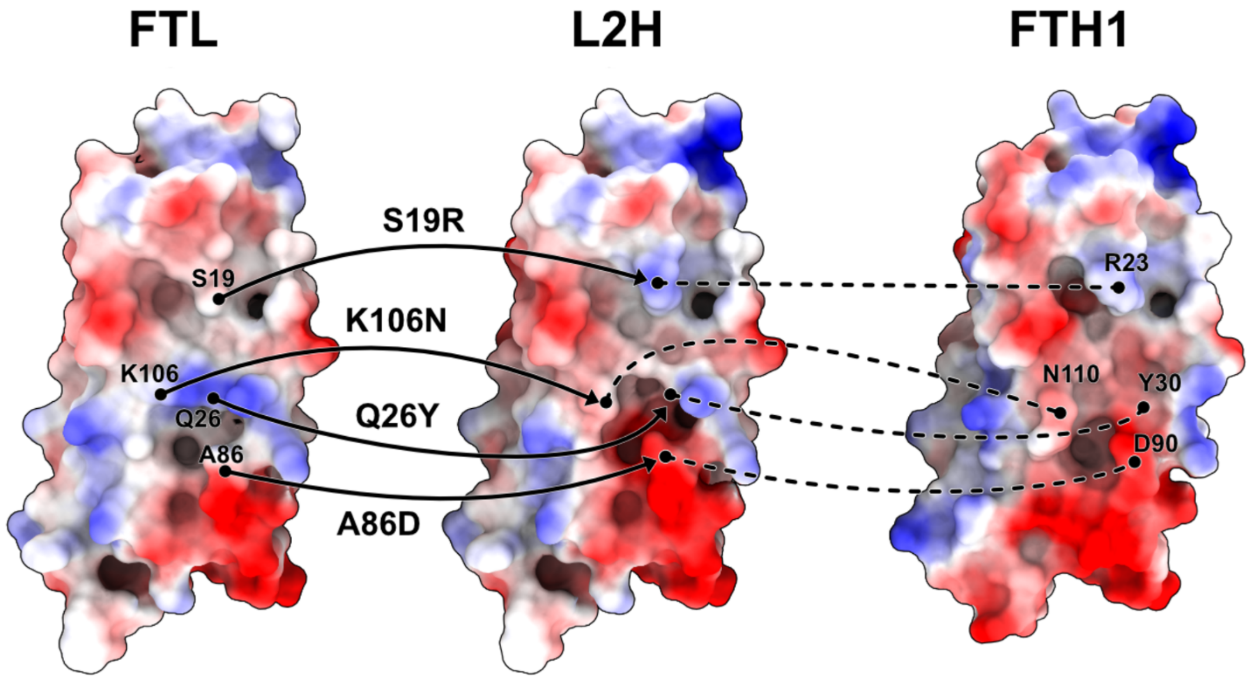

Supplementary Fig. 4 Visualization of the structure-guided design of the binding site for NCOA4 on the surface FTL. The panels depict the electrostatic surface of *WT*-FTL on the left, *WT*-FTH1 on the right, and the designed L2H mutant in the middle. Electrostatic surface potentials on the structures are colored red and blue for negative and positive charges, respectively, and white color represents neutral residues.

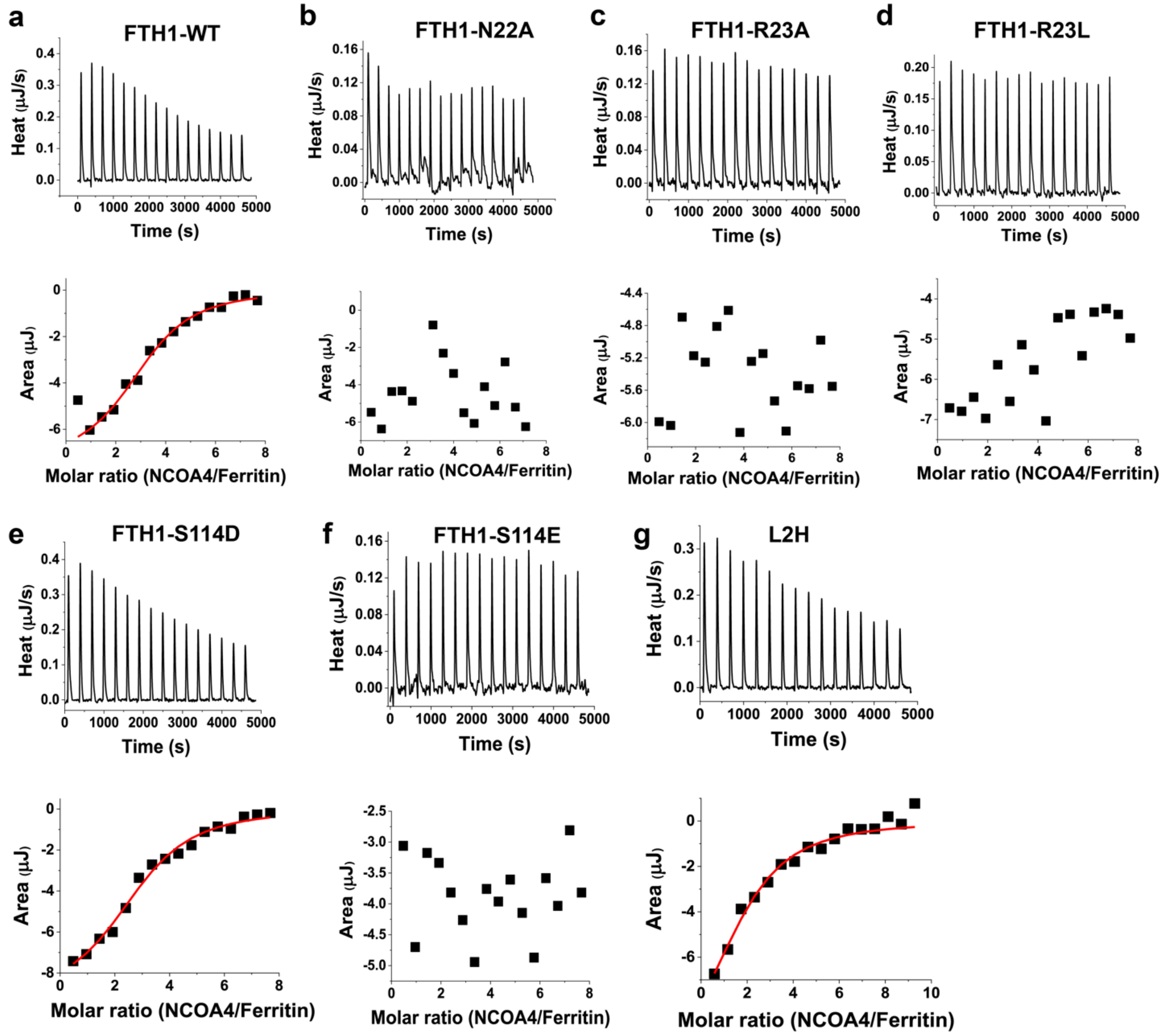

Supplementary Fig. 5 Compilation of raw ITC data and fit results. The top panels depict the instrument's power response measured over time upon consecutive injections. The bottom panels display the corresponding heats of reaction calculated from the integration of the area under each peak as a function of the molar ratio of NCOA4 to ferritin. When experimental heats of reaction are observed, the results were fitted to one independent binding site model (red curves). (**a**) FTH1-WT, (**b**) N22A, (**c**) R23A, (**d**) R23L, (**e**) S114D, (**f**) S114E, (**g**) L2H. Data from a representative experiment is presented (of two to three biologically independent experiments with similar results). Source data are provided as a Source Data file.

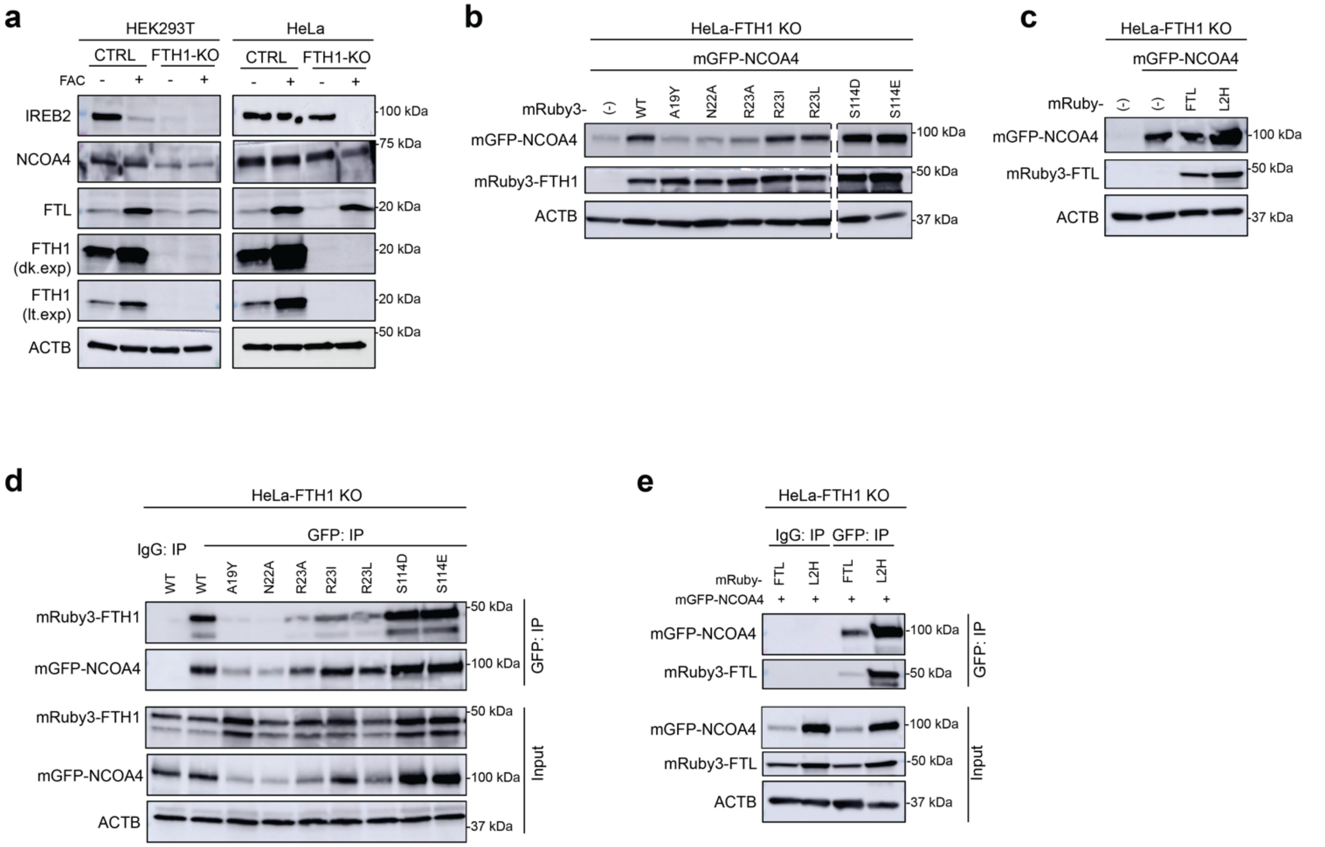

Supplementary Fig. 6 FTH1 and FTL mutants modulate affinity to NCOA4 in cells. (**a**) CRISPR/Cas9-mediated depletion of FTH1 expression in HEK293T and HeLa cells. Lysates from the indicated cells treated with 100 μM Ferric Ammonium Citrate (FAC, 16 h) were immunoblotted with the indicated antibodies. (**b**) Extracts from *FTH1* KO HeLa cells harboring stably expressed mGFP-NCOA4 and *WT* or mutant mRuby3-FTH1were immunoblotted with the indicated antibodies. (**c**) Extracts from HeLa-*FTH1* KO cells harboring stably expressed mGFP-NCOA4 and WT or mutant mRuby3-FTL were immunoblotted with the indicated antibodies. (**d**) Extracts from HeLa-*FTH1* KO cells harboring stably expressed mGFP-NCOA4 and *WT* or mutant mRuby3-FTH1 were immunoprecipitated with anti-GFP and immunoblotted with the indicated antibodies. (**e**) Extracts from HeLa-*FTH1* KO cells harboring stably expressed mGFP-NCOA4 and WT or mutant mRuby3-FTL were immunoprecipitated with anti-GFP and immunoblotted with the indicated antibodies. For all panels, a representative blot of two biologically independent experiments with similar results is presented. Uncropped blots are provided in Supplementary Fig. 7.

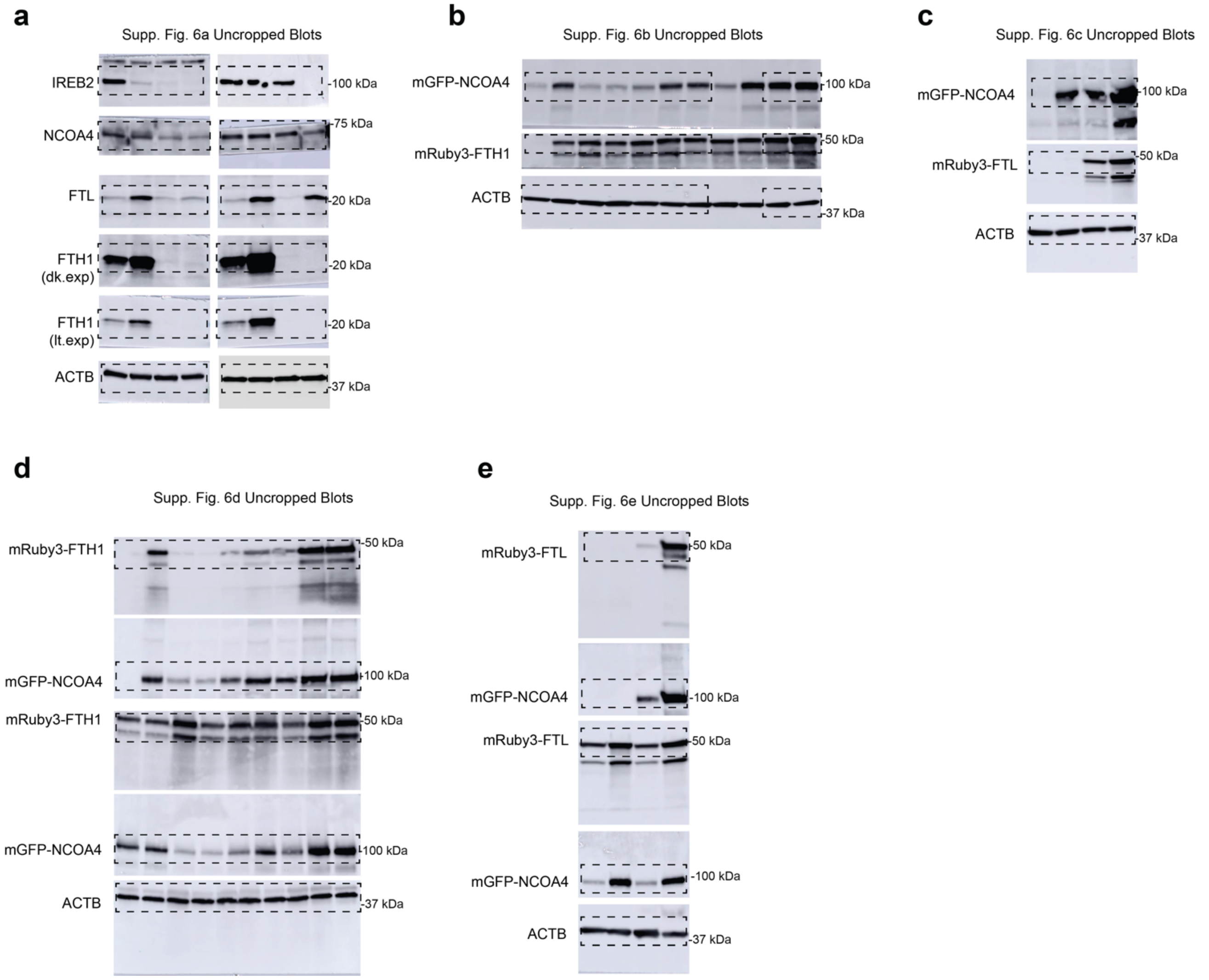

Supplementary Fig. 7 Uncropped western blots depicted in Supplementary Fig. 6. The position of each blot in Supplementary Fig. 6 is designated above the blot. The segments used in the figures are designated with a dashed frame. (**a**) Supp Fig. 6a uncropped blots. (**b**) Supplementary Fig. 6b uncropped blots. (**c**) Supplementary Fig. 6c uncropped blots. (**d**) Supplementary Fig. 6d uncropped blots. (**e**) Supplementary Fig. 6e uncropped blots.

**Supplementary Table 1**: **Cryo-EM data collection, refinement and validation statistics.**	
PDB and EMDB accession codes:	EMD-18658 [https://www.ebi.ac.uk/emdb/EMD-18658]<br>PDB: 8QU9 [https://doi.org/10.2210/pdb8qu9/pdb]
**Data collection and processing**	
Magnification	130k
Voltage (kV)	200
Electron exposure (e–/Å^2)	30
Defocus range (μm)	-0.5 to -2.0
Pixel size (Å)	0.89
Symmetry imposed	O
Initial particle images (no.)	83,195
Final particle images (no.)	33,749
Map resolution (Å)<br>FSC thresholds=0.5 / 0.143	3.21 / 2.88
Map resolution range (Å)	2.86 to 4.13
**Refinement**	
Initial model used (PDB code)	7RRP
Model resolution (Å)<br>FSC threshold=0.143	2.6
Model resolution range (Å)	
Map sharpening *B* factor (Å^2)	-95.7
Model composition	
Non-hydrogen atoms	1550
Protein residues	188
Ligands	Fe: 1
B factors (Å^2)	
Protein	-35.72
Ligand	-80.27
R.m.s. deviations	
Bond lengths (Å)	0.004
Bond angles (°)	0.796
Validation	
MolProbity score	1.32 (98th percentile)
Clashscore	5.93 (91st percentile)
Poor rotamers (%)	0
Ramachandran plot	
Favored (%)	100
Allowed (%)	0
Disallowed (%)	0
