# Supplementary material for: Structural basis for the intracellular regulation of ferritin degradation
Source: Nat Commun. 2024 May 7;15:3802. doi: 10.1038/s41467-024-48151-1 (PMC11076521; doi:10.1038/s41467-024-48151-1)
Supplement: Supplementary file 3 — Reporting Summary [file 41467_2024_48151_MOESM3_ESM.pdf]

nature portfolio

Corresponding author(s): Gabriel A. Frank and Joseph D. Mancias

Last updated by author(s): 2024-4-2

Reporting Summary

Nature Portfolio wishes to improve the reproducibility of the work that we publish. This form provides structure for consistency and transparency in reporting. For further information on Nature Portfolio policies, see our Editorial Policies and the Editorial Policy Checklist.

Statistics

For all statistical analyses, confirm that the following items are present in the figure legend, table legend, main text, or Methods section.

n/a	Confirmed	
☐	☒	The exact sample size (*n*) for each experimental group/condition, given as a discrete number and unit of measurement
☐	☒	A statement on whether measurements were taken from distinct samples or whether the same sample was measured repeatedly
☐	☒	The statistical test(s) used AND whether they are one- or two-sided<br>*Only common tests should be described solely by name; describe more complex techniques in the Methods section.*
☒	☐	A description of all covariates tested
☒	☐	A description of any assumptions or corrections, such as tests of normality and adjustment for multiple comparisons
☐	☒	A full description of the statistical parameters including central tendency (e.g. means) or other basic estimates (e.g. regression coefficient) AND variation (e.g. standard deviation) or associated estimates of uncertainty (e.g. confidence intervals)
☐	☒	For null hypothesis testing, the test statistic (e.g. *F*, *t*, *r*) with confidence intervals, effect sizes, degrees of freedom and *P* value noted<br>*Give P values as exact values whenever suitable.*
☒	☐	For Bayesian analysis, information on the choice of priors and Markov chain Monte Carlo settings
☒	☐	For hierarchical and complex designs, identification of the appropriate level for tests and full reporting of outcomes
☒	☐	Estimates of effect sizes (e.g. Cohen's *d*, Pearson's *r*), indicating how they were calculated

Our web collection on statistics for biologists contains articles on many of the points above.

Software and code

Policy information about availability of computer code

Data collection	Cryo-EM: EPU (v3.4.0), ITC: ITCRun (v. 3.8.0.18730)
Data analysis	Cryo-EM: Relion (version: 3.1.1-commit-879ea4), CTFFind4 (4.1.14)<br>ITC: NanoAnalyze (v3.12.5)<br>IP and Immunofluorescence: GraphPad Prism (v10.2.0), ImageJ (v1.54h)

For manuscripts utilizing custom algorithms or software that are central to the research but not yet described in published literature, software must be made available to editors and reviewers. We strongly encourage code deposition in a community repository (e.g. GitHub). See the Nature Portfolio guidelines for submitting code & software for further information.

Data

Policy information about availability of data

All manuscripts must include a data availability statement. This statement should provide the following information, where applicable:

- Accession codes, unique identifiers, or web links for publicly available datasets
- A description of any restrictions on data availability
- For clinical datasets or third party data, please ensure that the statement adheres to our policy

The Cryo-EM map and resulting molecular model are available in the Electron Microscopy Data Bank (EMDB) accession number EMD-18658 [https://www.ebi.ac.uk/emdb/EMD-18658] and the Protein Data Bank (PDB) accession code 8QU9 [https://doi.org/10.2210/pdb8qu9/pdb], respectively. The raw ITC data and western blots

data are provided in the supplementary material and source data. The optical microscopy data is available on figshare, DOI: available after paper submission (as per instructions on Nature figshare uploading tool). Source data are provided with this paper.

Research involving human participants, their data, or biological material

Policy information about studies with human participants or human data. See also policy information about sex, gender (identity/presentation), and sexual orientation and race, ethnicity and racism.

Reporting on sex and gender	NA
Reporting on race, ethnicity, or other socially relevant groupings	NA
Population characteristics	NA
Recruitment	NA
Ethics oversight	NA

Note that full information on the approval of the study protocol must also be provided in the manuscript.

Field-specific reporting

Please select the one below that is the best fit for your research. If you are not sure, read the appropriate sections before making your selection.

☒ Life sciences ☐ Behavioural & social sciences ☐ Ecological, evolutionary & environmental sciences

For a reference copy of the document with all sections, see nature.com/documents/nr-reporting-summary-flat.pdf

Life sciences study design

All studies must disclose on these points even when the disclosure is negative.

Sample size	No statistical method was used to predetermine sample size, as the magnitude of the effect sizes were unknown. As reference, the sample sizes were guided on the basis of similar published studies (PMID: 26436293). All experiments were included with multiple biological replicates based on previous experiences (PMID: 26436293).
Data exclusions	No samples were excluded from analysis.
Replication	All results were tested and confirmed with at least two independent experiments.
Randomization	No method of randomization was applied. Samples were organized into groups based on whether they were wild type or mutant FTH1 or FTL.
Blinding	No blinding assessment was performed for structural studies. For quantification of IF-based colocalization experiments, the investigator was blinded to the group during quantification.

Reporting for specific materials, systems and methods

We require information from authors about some types of materials, experimental systems and methods used in many studies. Here, indicate whether each material, system or method listed is relevant to your study. If you are not sure if a list item applies to your research, read the appropriate section before selecting a response.

Materials & experimental systems

n/a	Involved in the study	
☐	☒	Antibodies
☐	☒	Eukaryotic cell lines
☒	☐	Palaeontology and archaeology
☒	☐	Animals and other organisms
☒	☐	Clinical data
☒	☐	Dual use research of concern
☒	☐	Plants

Methods

n/a	Involved in the study	
☒	☐	ChIP-seq
☒	☐	Flow cytometry
☒	☐	MRI-based neuroimaging

Antibodies

Antibodies used

The following antibodies were used:
NCOA4 (Santa Cruz Biotechnology, sc-373739, 1:100),
FTH1 (Cell Signaling Technology (CST), 4393, 1:2,000),
ACTB (Sigma, A5441, 1:5,000),
anti-DYKDDDDK (CST, 14793, 1:1000);
HA-Tag (CST, 3724, 1:1000),
cMyc (Invitrogen, MA1-980, 1:1000),
GFP (CST, 2956, 1:1000)
Secondary antibodies were as follows:
anti-rabbit IgG (H+L) HRP conjugate (Thermo, 31460, 1:5000),
anti-mouse IgG (H1L) HRP conjugate (Promega, W4021, 1:5000),
anti-mouse IgG Fc cross-absorbed HRP conjugate (Invitrogen, 31439, 1:5000)

Validation

Antibodies for NCOA4 and FTH1 were validated as follows: For FTH1 antibody, specificity was confirmed using FTH1 knockouts as displayed in Supplementary Fig. 6a. For NCOA4, specificity was determined using NCOA4 knockout cell lines as described in PMID: 35771492 and PMID: 26436293.

Eukaryotic cell lines

Policy information about cell lines and Sex and Gender in Research

Cell line source(s)

HEK293T and HeLa cells were obtained from ATCC

Authentication

Cell lines were maintained in a centralized cell bank, authenticated by assessment of cell morphology as well as short tandem repeat fingerprinting.

Mycoplasma contamination

Cells were routinely inspected for Mycoplasma contamination using PCR and were all negative.

Commonly misidentified lines
(See ICLAC register)

No commonly misidentified lines were used.

Plants

Seed stocks

Report on the source of all seed stocks or other plant material used. If applicable, state the seed stock centre and catalogue number. If plant specimens were collected from the field, describe the collection location, date and sampling procedures.

Novel plant genotypes

Describe the methods by which all novel plant genotypes were produced. This includes those generated by transgenic approaches, gene editing, chemical/radiation-based mutagenesis and hybridization. For transgenic lines, describe the transformation method, the number of independent lines analyzed and the generation upon which experiments were performed. For gene-edited lines, describe the editor used, the endogenous sequence targeted for editing, the targeting guide RNA sequence (if applicable) and how the editor was applied.

Authentication

Describe any authentication procedures for each seed stock used or novel genotype generated. Describe any experiments used to assess the effect of a mutation and, where applicable, how potential secondary effects (e.g. second site T-DNA insertions, mosiacism, off-target gene editing) were examined.
